# Mountain lions prey selectively on prion-infected mule deer

**DOI:** 10.1098/rsbl.2009.0742

**Published:** 2009-10-28

**Authors:** Caroline E. Krumm, Mary M. Conner, N. Thompson Hobbs, Don O. Hunter, Michael W. Miller

**Affiliations:** 1 Colorado Division of Wildlife, Wildlife Research Center, Fort Collins, CO 80526-2097, USA; 2 Graduate Degree Program in Ecology, Colorado State University, Fort Collins, CO 80523, USA; 4 Natural Resource Ecology Laboratory, Colorado State University, Fort Collins, CO 80523, USA; 3 Department of Wildland Resources, Utah State University, Logan, UT 84322-5230, USA; 5 United States Fish and Wildlife Service, Fort Collins, CO 80526, USA

**Keywords:** chronic wasting disease, predation, prion, *Puma concolor*, selection, vulnerability

## Abstract

The possibility that predators choose prey selectively based on age or condition has been suggested but rarely tested. We examined whether mountain lions (*Puma concolor*) selectively prey upon mule deer (*Odocoileus hemionus*) infected with chronic wasting disease, a prion disease. We located kill sites of mountain lions in the northern Front Range of Colorado, USA, and compared disease prevalence among lion-killed adult (≥2 years old) deer with prevalence among sympatric deer taken by hunters in the vicinity of kill sites. Hunter-killed female deer were less likely to be infected than males (odds ratios (OR) = 0.2, 95% confidence intervals (CI) = 0.1–0.6; *p* = 0.015). However, both female (OR = 8.5, 95% CI = 2.3–30.9) and male deer (OR = 3.2, 95% CI = 1–10) killed by a mountain lion were more likely to be infected than same-sex deer killed in the vicinity by a hunter (*p* < 0.001), suggesting that mountain lions in this area actively selected prion-infected individuals when targeting adult mule deer as prey items.

## 1. Introduction

Theoretical models and some empirical evidence suggest that predators select prey based in part on their vulnerability (Emlen 1966; MacArthur & Pianka 1966; Curio 1976; Temple 1987). Selecting prey in poor condition may conserve energy or reduce the risk of injury (Mech 1970; Ackerman *et al*. 1984; Pierce *et al*. 2000). Thus, a prevailing idea in ecology is that predators capture young, old, sick, weak, injured or inexperienced individuals from prey populations in higher than expected proportions (Errington 1946; Slobodkin 1968; Curio 1976). Despite its wide acceptance, this idea rarely has been tested.

Mountain lions (*Puma concolor*) are ambush predators (Hornocker 1970; Logan & Sweanor 2001). Young and/or solitary deer (*Odocoileus* spp.) are most vulnerable to mountain lion predation (Hornocker 1970; Logan & Sweanor 2001). However, previous studies have not examined whether diseased deer are more vulnerable to or selected by mountain lions.

Chronic wasting disease (CWD) (Williams & Young 1980) is a naturally occurring prion disease of North American deer. Simulations suggest that selectively removing infected individuals via test-and-cull or predation could reduce prevalence (Gross & Miller 2001), and thus would be valuable in disease control. Clinical signs of CWD are progressive and include poor body condition, altered behaviour, incoordination and periods of somnolence (Williams & Young 1980). It follows that infected deer may be more susceptible to predation than uninfected individuals because they are less cautious and less able to recognize and respond to threats (Williams & Young 1980; Chase-Topping *et al*. 2005; Krumm *et al*. 2005; Miller *et al*. 2008). Here, we evaluated whether mountain lions are more likely to prey upon prion-infected mule deer (*Odocoileus hemionus*) than upon uninfected individuals.

## 2. Material and methods

Nine captured mountain lions older than one year were fitted with GPS collars in the northern Front Range of Colorado, USA. GPS data were obtained through remote download. We used cluster analysis of greater than or equal to three location data points within 200 m over a 24 h period to determine the locations of possible kill sites (Anderson & Lindzey 2003). Once a cluster was identified, we used its centre in attempting to locate the kill site. If the prey item was a mule deer and appropriate tissues were available, samples were tested for prion infection. We also collected samples from other mountain-lion-killed mule deer carcasses found in the study area during the same time period. Prion diagnostic methods were as described in Miller & Conner (2005).

For comparison to lion-killed mule deer, we used data from mule deer sampled in the vicinity of identified lion-kill sites (hereafter referred to as ‘vicinity-sampled’). We defined vicinity as less than or equal to 3 km radius of a lion-kill site because from a previous study 86 percent of movements made by local mule deer were less than or equal to 3 km during non-migratory periods (Conner & Miller 2004). This approximately 28 km^2^ area represented local prion infection risk. We only included vicinity samples from the same overall time period as the lion-killed samples. The source of vicinity samples was mule deer killed by hunters and tested using the same diagnostic methods as above.

To assess the differential probability of mountain lion predation, we compared the odds of infection (odds ratio (OR)) among lion-killed deer to that among vicinity-sampled deer. We used data from lion-killed deer that had greater than or equal to three vicinity samples in these analyses. Because prevalence in mule deer differs by age, sex and population (Miller & Conner 2005), we only used data for adult (≥2 years of age) deer and factored sex and population influences into our analyses. We estimated the prevalence among lion-killed deer and vicinity-sampled deer using least-squares means and their 95 per cent confidence intervals (CI) from a generalized linear mixed model approach (Proc GLIMMIX; SAS Institute 2008). We used a logistic model with the explanatory variables (fixed effects) sex, kill type (lion- or vicinity-) and sex × kill type; we included source (the cluster of kills in the vicinity of a lion kill) as a random effect to account for spatial heterogeneity. Among the adult-lion-killed deer (10 infected and 31 uninfected) that had been assigned to age groups (2–4 years, 5–7 years or >8 years old) by examining dentition, we also compared the occurrence by infection status across three age classes post hoc using a Fisher exact 2 × 3 contingency table.

## 3. Results

From January 2003 to July 2006, we found prey remains at 108 kill sites, including 62 mule deer carcasses. In all, there were 54 lion-killed deer carcasses that were greater than or equal to 2 years of age, had suitable tissue available and had greater than or equal to three associated vicinity kills.

**Figure 1.**
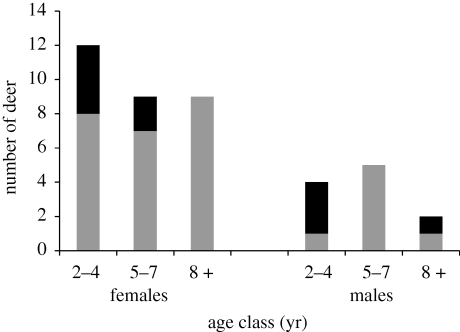
Numbers of adult (≥2 year old) prion-infected and uninfected mule deer (*n* = 41) killed by mountain lions, assigned to age classes representing young (2–4 years), middle-aged (5–7 years) or older (>8 years) individuals. The age distribution of infected deer resembled the patterns reported elsewhere (Miller & Conner 2005; Miller *et al*. 2008). Black shaded box, infected; grey shaded box, uninfected.

Hunter-killed female deer were less likely (*p* = 0.015) to be infected than males (OR = 0.2, 95% CI = 0.1–0.6; table 1), but both female (OR = 8.5, 95% CI = 2.3–30.9) and male deer (OR = 3.2, 95% CI = 1–10) killed by a mountain lion were more likely to be infected than same-sex deer killed in the vicinity by a hunter (table 1). Sex and kill type (lion versus vicinity) were significant fixed effects, but their interaction was not (table 2). The estimate for the random effect (source) was zero. Among 41 lion-killed deer that we could age to the nearest year, infected individuals tended to be younger than uninfected individuals (Fisher exact 2 × 3 contingency table *p* = 0.1; figure 1).

**Table 1.** Estimated prevalence of prion infection among mountain-lion-killed adult (≥2 years old) mule deer and among sympatric adult deer sampled in the vicinity of lion kills.

	lion-killed deer				deer sampled in vicinity			
sex	*n* sampled	*n* positive	prevalence^a^	95% CI	*n* sampled	*n* positive	prevalence	95% CI
female	37	7	0.19	0.09–0.35	149	4	0.03	0.01–0.07
male	17	5	0.29	0.13–0.54	163	19	0.12	0.08–0.18

^a^Prevalence and its 95% CI are back transformed least-square means estimates from Proc GLIMMIX (SAS Institute 2008) for a model having kill type, sex, and kill type × sex as fixed effects. Population source was a random effect, estimated as 0.

**Table 2.** Fixed effects statistics from a model evaluating prion infection patterns among adult (≥2 years old) mule deer.

	numerator	denominator		
effect	d.f.	d.f.	*F*	*p*
kill type^a^	1	360	13.91	<0.001
sex	1	360	5.93	0.015
kill type × sex	1	360	1.25	0.264

^a^Kill type was deer killed by mountain lions or deer killed by hunters in the vicinity of lion kills.

## 4. Discussion

Adult mule deer killed by mountain lions were more likely to be prion-infected than were deer killed more randomly in sympatric populations, suggesting that mountain lions were selecting for infected individuals when they targeted adult deer. In roughly the same geographical area where we sampled mountain-lion-kill sites, Krumm *et al*. (2005) found that deer killed in vehicle collisions had the odds of prion infection (OR = 2.4, 95% CI = 1.4–4.1) similar to those estimated from our data. However, a separate cohort study of mule deer survival at Table Mesa (also located within our study area) revealed that prion-infected deer had a much greater risk (3.7×, 95% CI = 1.1–12.5) of being killed by mountain lions than by vehicles, even though uninfected deer in this area were equally likely to be killed by either mountain lions or vehicles (relative risk = 0.6, 95% CI = 0.2–2.4; Miller *et al*. 2008). From the observations gathered across several studies, we hypothesize that although much of the ‘selection’ we observed may be attributed to infected mule deer being less vigilant or fit and thus relatively vulnerable to ‘attack’ of one kind or another, mountain lions may also learn to recognize and more actively target diseased deer.

Other studies indicate that coursing predators like wolves (*Canis lupus*) and coyotes (*C. latrans*) select prey disproportionately if they appear impaired by malnutrition, age or disease (Crisler 1956; Mech 1970; Gese & Grothe 1995; Lingle & Wilson 2001). Although a stalking predator might not be expected to be as selective as a coursing predator, mountain lions apparently can be as selective—relative to the availability of different age and condition categories of prey—as coyotes (Pierce *et al*. 2000). The subtle behaviour changes in prion-infected deer may be better signals of vulnerability than body condition, and these cues may occur well before body condition noticeably declines (Williams & Young 1980; Chase-Topping *et al*. 2005; Krumm *et al*. 2005; Miller *et al*. 2008). The tendency for infected-lion-killed deer to be relatively young adults compared to uninfected-lion-killed deer (figure 1) suggests that such cues were sufficiently strong to draw attention to (or increase vulnerability of) individuals outside the age classes typically targeted by mountain lions hunting in this area.

Intuitively, we expect predators to be more successful in capturing animals that are slow or less alert. The ‘sanitation effect’ of predators selecting weak individuals over prime, healthy specimens (Leopold 1933; Mech 1970) has been documented in several studies (Mech 1966; Kolenosky 1972; Schaller 1972). Although theory suggests that removing infected animals could ‘sanitize’ and slow the rate of prion transmission (Gross & Miller 2001), prevalence can be remarkably high in mule deer populations preyed upon by mountain lions (Miller *et al*. 2008). Prion transmission among deer can occur via several mechanisms, including indirect transmission from exposure to prions in the environment (Miller *et al*. 2004). We observed that mountain lions typically consumed greater than 85 percent of a deer carcass, often including brain tissue, and this may be beneficial in decreasing prion contamination at kill sites. However, the extent to which selective predation by mountain lions alters the dynamics of prion disease epidemics in natural mule deer populations remains unclear (Miller *et al*. 2008).
